# An Arabidopsis mutant with high operating efficiency of Photosystem II and low chlorophyll fluorescence

**DOI:** 10.1038/s41598-017-03611-1

**Published:** 2017-06-12

**Authors:** Niels van Tol, Martijn Rolloos, Dieuwertje Augustijn, A. Alia, Huub J. de Groot, Paul J. J. Hooykaas, Bert J. van der Zaal

**Affiliations:** 1 Institute of Biology Leiden, Faculty of Science, Leiden University, Sylviusweg 72, 2333 BE Leiden, The Netherlands; 2 BioSolar Cells, P.O. Box 98, 6700 AB Wageningen, The Netherlands; 3 Leiden Institute of Chemistry, Faculty of Science, Leiden University, Einsteinweg 55, 2333 CC Leiden, The Netherlands

## Abstract

The overall light energy to biomass conversion efficiency of plant photosynthesis is generally regarded as low. Forward genetic screens in Arabidopsis have yielded very few mutants with substantially enhanced photochemistry. Here, we report the isolation of a novel Arabidopsis mutant with a high operating efficiency of Photosystem II (φPSII) and low chlorophyll fluorescence from a library of lines harboring T-DNA constructs encoding artificial transcription factors. This mutant was named Low Chlorophyll Fluorescence 1 (LCF1). Only a single T-DNA insertion was detected in LCF1, which interrupted the expression of the full length mRNA of the gene At4g36280 (*MORC2*). We demonstrate that the high φPSII and low levels of chlorophyll fluorescence were due to a decrease in PSII:PSI ratio. Although LCF1 plants had decreased rosette surface area and biomass under normal growth conditions, they contained more starch per gram fresh weight. The growth defect of LCF1 was alleviated by low light and short day conditions, and growth could even be enhanced after a period of dark-induced senescence, showing that the plant can utilize its excess photosynthetic conversion capacity as a resource when needed.

## Introduction

Photosynthesis is the process that harvests energy from sunlight and fixes it as chemical energy. The light is absorbed by chlorophyll molecules that are associated with the antenna complexes of Photosystems I and II (PSI and PSII) in the thylakoid membranes of chloroplasts, which results in the photoexcitation and subsequent charge separation of the reaction center chlorophylls P700 and P680, respectively. The water-plastoquinone A oxidoreductase activity of PSII, referred to as photochemistry, initiates a linear flow of photoexcited electrons from PSII to the final electron acceptor NADP, thus generating NADPH, and drives the synthesis of ATP by chemiosmotic coupling. NADPH and ATP are used in the Calvin-Benson cycle for CO_2_ fixation by the enzyme complex RuBisCo. The resulting carbohydrate product is partitioned to different parts and processes of the plant. Photosynthesis is therefore considered the driving force behind plant productivity. However, the overall light energy to biomass conversion efficiency of photosynthesis is generally regarded as remarkably low^1^. Photosynthesis has therefore become a primary target for the improvement of crop yield^2^, and the genetic modification of photosynthesis has received a considerable amount of attention^3–6^.

Apart from photochemistry, photoexcitation events in PSII reaction centers can lead to the emission of chlorophyll fluorescence (CF), and to the dissipation of excess excitation energy through various processes that are together named non-photochemical quenching (NPQ)^7^. CF can relatively easily be observed and quantified with a fluorescence camera upon illumination of a plant with actinic light. Photochemistry and NPQ are both quenchers of CF, and changes in the rates of photochemistry and NPQ are therefore proportionally reflected by changes in CF levels. CF imaging with Pulse-Amplitude-Modulation (PAM) equipment therefore allows for the relatively accurate quantification of photochemistry, the rate of linear electron transport and of NPQ^8^. In addition, CF imaging allows for the relatively easy quantification of the maximum quantum efficiency of PSII photochemistry (F_v_/F_m_) and of the operating light use efficiency of Photosystem II (φPSII)^8^. The latter parameter is considered to be an accurate measure for linear electron transport rate and the rate of CO_2_ fixation in both C3 and C4 plant species^8^. In principle, CF imaging can thus be used to accurately estimate the overall photosynthetic performance of plants, thereby making the isolation of photosynthesis mutants from populations of plants a feasible approach.

Several published and ongoing studies have used φPSII as a means of assessing variation in the photosynthetic efficiency of natural and mutant Arabidopsis accessions. For the natural Arabidopsis accessions these studies have been carried out on a large scale^9, 10^, but very little natural variation in φPSII has been found^10^. Surprisingly, no large scale mutant screens with φPSII as a selection criterion have been described in literature. Very recently, after first using F_v_/F_m_ as a selection criterion, insertion mutants of the Arabidopsis gene *HPE1* (At1g70200) were described which exhibited substantially higher φPSII than Col-0^11^. To our knowledge, these are the only recessive knock-out mutants with this phenotype that have been described in literature.

In our lab, we have established zinc finger artificial transcription factor (ZF-ATF)-mediated genome interrogation^12^ as a tool for generating novel Arabidopsis mutants with enhanced traits^13–15^. We have used genome interrogation to investigate whether an innate capacity to perform more efficient photosynthesis can be artificially invoked in Arabidopsis by the large scale distortion of gene expression patterns. In our setup, ZF-ATFs consist of an array of three zinc fingers (3F) fused to the transcriptional activator protein VP16^13^. Each ZF recognizes a cognate 3 base pair (bp) 5′-GNN-3′ consensus DNA sequence (‘N’ can be any of the DNA bases)^16^. There are 16 possible 5′-GNN-3′ binding ZFs, which can theoretically be assembled into 256 two finger (2F) and 4096 3F combinations. Arabidopsis plants are transformed with the artificial 3F-VP16 encoding gene construct under control of the promoter of the Arabidopsis *RPS5a* gene^17^, which is predominantly active in embryonic and meristematic tissue. Due to the relatively short DNA recognition sequence, each 3F-VP16 fusion on average has approximately 1000 binding sites in the 130 Mbp Arabidopsis genome, where it can influence the expression of nearby genes *in trans* and in a dominant manner through the activity of VP16. Introducing a single artificial gene in an otherwise wild type genome thus allows for the drastic perturbation of genome-wide gene expression patterns, and the potential induction of (novel) traits of interest.

In the present study, we have screened a library of Arabidopsis lines harboring 3F-VP16 encoding T-DNA constructs for lines with enhanced φPSII. Through this screen, a novel recessive Arabidopsis mutant with high φPSII and low CF was isolated, which was named Low Chlorophyll Fluorescence 1 (LCF1). The phenotypes of LCF1 were linked to a single T-DNA insertion in the locus At4g36280 (*MORC2*). Furthermore, we describe the CF and growth characteristics of LCF1, and propose a model integrating these data.

## Results

### Genome interrogation library construction and isolation of LCF1

**Figure 1:**
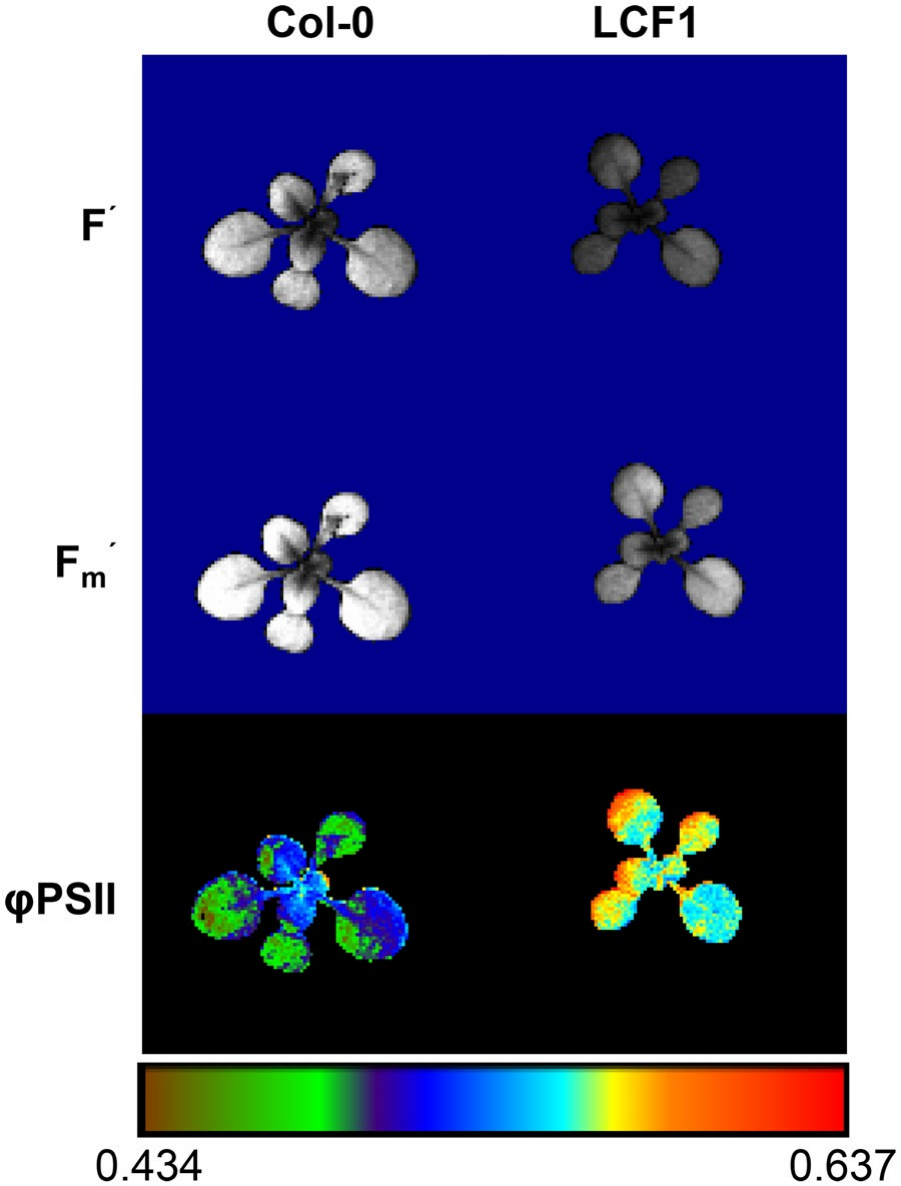
Overview of the chlorophyll fluorescence (CF) characteristics of Col-0 and LCF1 plants. False color images of steady state CF (F′), maximal CF (F_m_′) and the operating light use efficiency of Photosystem II (φPSII), all at 200 μmol m^−2^ s^−1^ actinic light.

In order to investigate whether genome interrogation can be used to generate novel plant lines with enhanced photosynthetic properties, a library of Arabidopsis plant lines harboring 3F-VP16 encoding T-DNA constructs was screened for significant changes in φPSII compared to the wild type Col-0 using CF imaging. Briefly, the genome interrogation library was constructed by floral dip transformation of Col-0 plants with a library of pRF-VP16-Kana binary vectors harboring these T-DNA constructs^13^. The library consisted of seeds that were harvested from primary transformants (T1), and are therefore referred to as the second generation of transformants (T2). Similarly, the progeny of the secondary generation of transformants is referred to as the tertiary generation (T3), and so on. The library had approximately 3500 different genome interrogation constructs represented as T-DNA insertions in a total of 4278 lines. Using 200 μmol m^−2^ s^−1^ of actinic light, a population of genome interrogation T2 plants was screened for φPSII values that deviated strongly from Col-0. One mutant individual with high φPSII, low steady state CF and low maximal CF in the light was isolated (Fig. 1). As the T3 offspring of this individual did not segregate for the phenotype, the mutant plant line was named LCF1. Through PCR analysis and sequencing with T-DNA specific primers, the T-DNA insert in LCF1 was demonstrated to encode a ZF-ATF with a 3F DNA binding domain theoretically recognizing the 9 bp sequence GCG-GTG-GCG. We transformed Col-0 plants with a 3F-VP16 encoding T-DNA construct which was reconstituted with the 3F encoding sequence identified in LCF1, but these did not display high φPSII, demonstrating that the LCF1 phenotype was not triggered *in trans* by the 3F-VP16 fusion. In addition, when LCF1 (♂) was crossed with Col-0 (♀) only one quarter of their F2 offspring displayed the high φPSII phenotype (Fig. S1), indicative of a recessive mutation. Altogether these observations showed that expression of the 3F-VP16 fusion protein encoded by the T-DNA construct is not sufficient to induce the LCF1 phenotype and that the phenotypical characteristics of LCF1 are not a direct consequence of genome interrogation. Rather, the observations suggested that they result from a recessive mutation, possibly caused by a T-DNA insertion.

### Genetic analysis of LCF1

**Figure 2:**
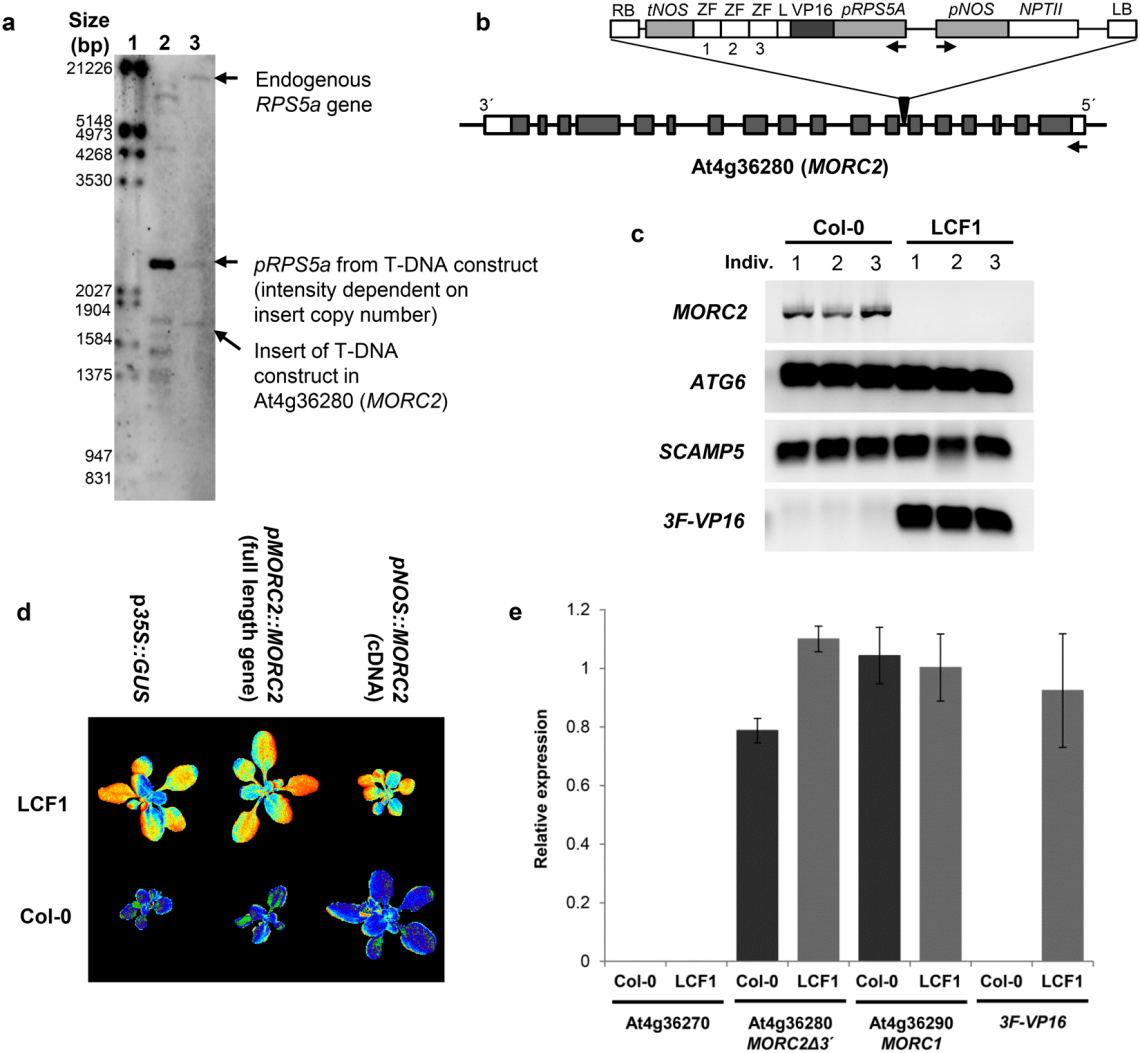
Genetic analysis of LCF1. (**a**) Southern blot on *Nco*I digested genomic DNA (gDNA) of empty vector control plants (Col-0 transformed with pRF-VP16-Kana not containing a 3F fragment; lane 2) and LCF1 (lane 3). Lane 1 contains DIG-labelled DNA ladder. The DIG-labelled probe was expected to hybridize with fragments of the promoter of the endogenous *RPS5a* gene (10695 bp fragment), the *RPS5a* promoter (p*RPS5a*) from the T-DNA construct (2590 bp fragment with intensity depending on the copy number of the insert), and the (3F)-VP16 fusion encoding part of each T-DNA insert in the genome (fragments of ≥~1500 bp for LCF1 and ≥~800 bp for the empty vector control without 3F). (**b**) Map of the 3F-VP16 encoding T-DNA construct in *MORC2* (At4g36280) of LCF1. *MORC2* is presented to scale. Exons are presented as grey boxes. 5′ and 3′ untranslated regions (UTRs) are presented as white boxes. The T-DNA insert is not presented to scale. (**c**) RT-PCR analysis of *MORC2* expression in Col-0 and LCF1 plants. The genes *ATG6* (At3g61710) and *SCAMP5* (At1g32050) serve as a controls for gene expression. Expression of the 3F-VP16 construct in LCF1 was verified as a positive control. (**d**) False color φPSII images of Col-0 and LCF1 plants (T1 generation; obtained through selection with PPT) harboring T-DNA constructs with either the *GUS* gene under control of the CaMV *35S* promoter (*p35S::GUS*), the full length genomic sequence of *MORC2* under control of its own promoter and terminator sequences (p*MORC::MORC2*) to assay for complementation, or the cDNA encoding MORC2 under control of the *NOS* promoter and terminator sequences (*pNOS::MORC2*) to assay for complementation. The variation in size among the transformants was due to herbicide selection and subsequent transfer from selection medium to soil. (**e**) RT-qPCR analysis of the expression of the 5′ end of *MORC2* (*MORC2Δ3*′) and of the two neighboring genes At4g36270 and At4g36290 (*MORC1*). Expression of the 3F-VP16 construct in LCF1 was quantified as a positive control. Relative expression values were obtained by normalizing to the expression values of the genes *ATG6* and *SCAMP5*. Error bars represent SEM values (n = 4). The presented data are the average of two technical replicates.

By means of Southern blotting, only one copy of the 3F-VP16 encoding T-DNA construct was detected in the genome of LCF1 (Fig. 2a). By means of Thermal Asymmetric Interlaced PCR (TAIL-PCR) the integration site of the T-DNA construct (Fig. 2b) was demonstrated to be the 6^th^ intron of the gene *MORC2* (At4g36280; chromosomal position 17165331–17169375 bp), which was confirmed by PCR analysis with a combination of T-DNA and *MORC2* specific primers. While full length *MORC2* mRNA was readily detected by RT-PCR analysis in Col-0 plants, no such transcripts were detected in LCF1 plants (Fig. 2c). In that respect, insertion of the 3F-VP16 encoding T-DNA construct in *MORC2* might have resulted in a functional knock-out of the gene. As expected, mRNA corresponding to the 3F-VP16 fusion protein encoded by the T-DNA insert was expressed in LCF1 (Fig. 2c and e). When the LCF1 phenotype would be a *MORC2* knock-out, we reasoned that it should be possible to find plants with a high φPSII phenotype among the progeny of the 15 available SALK T-DNA knock-out lines for *MORC2* of the Nottingham Arabidopsis Stock Center (Table 1). However, no LCF1-like phenotypes were detected in any of the SALK lines, indicating that a knock-out of *MORC2* is not sufficient to mimic LCF1. In addition to that, φPSII of LCF1 plants could not be restored to wild type levels by ectopic overexpression of the full length *MORC2* gene, nor by a recombinant version containing the complete coding sequence (Fig. 2d). To establish genetic linkage between the phenotype of LCF1 and *MORC2*, LCF1 was crossed with the accession Ler-0 to establish a mapping population of F2 hybrid plants. From this population, 110 individuals with high φPSII and low CF were genotyped by PCR analysis using primers for the indel marker 4-AL022141-9227, which is located close to *MORC2* at the chromosomal position 17148559 bp, a physical distance of less than 20,000 bp and thus within a genetic distance of 1 cM. Only two out of 220 chromosomes were found the be recombinant, indicating that the genetic element linked to the phenotype of LCF1 was indeed located very close to this marker, making the T-DNA insertion locus in *MORC2* a prime candidate. By RT-qPCR analysis it was further investigated whether the T-DNA insertion in *MORC2* somehow modified expression of the directly flanking genes, At4g36270 and At4g36290 (*MORC1*). For *MORC1*, no significant differences compared to Col-0 were observed and the non-expressed gene At4g36270 was also not expressed in LCF1 (Fig. 2e). By using RT-qPCR analysis with primers specific for the 5′ part of the *MORC2* transcript, upstream of the T-DNA insertion (Fig. 2b), we found that a truncated *MORC2* transcript is still present in LCF1 (Fig. 2e). It might be that the LCF1 phenotype is triggered by the expression of a truncated MORC2 protein, but the recessive nature of the mutation would then suggest that the phenotype is dosage dependent and becomes apparent only in the homozygous state. Alternatively, another recessive mutation might have occurred close by.

**Table 1:** The SALK T-DNA insertion lines for the genomic locus At4g36280 that were used in this study.

NASC Code	Accession name
N521267	SALK_021267
N572456	SALK_072456
N572774	SALK_072774
N585402	SALK_085402
N585404	SALK_085404
N589534	SALK_089534
N623179	SALK_123179
N635895	SALK_135895
N636332	SALK_136332
N636502	SALK_136502
N636598	SALK_136598
N636599	SALK_136599
N645794	SALK_145794
N669832	SALK_072774C
N674058	SALK_021267C

### LCF1 plants have high φPSII and low chlorophyll fluorescence

**Figure 3:**
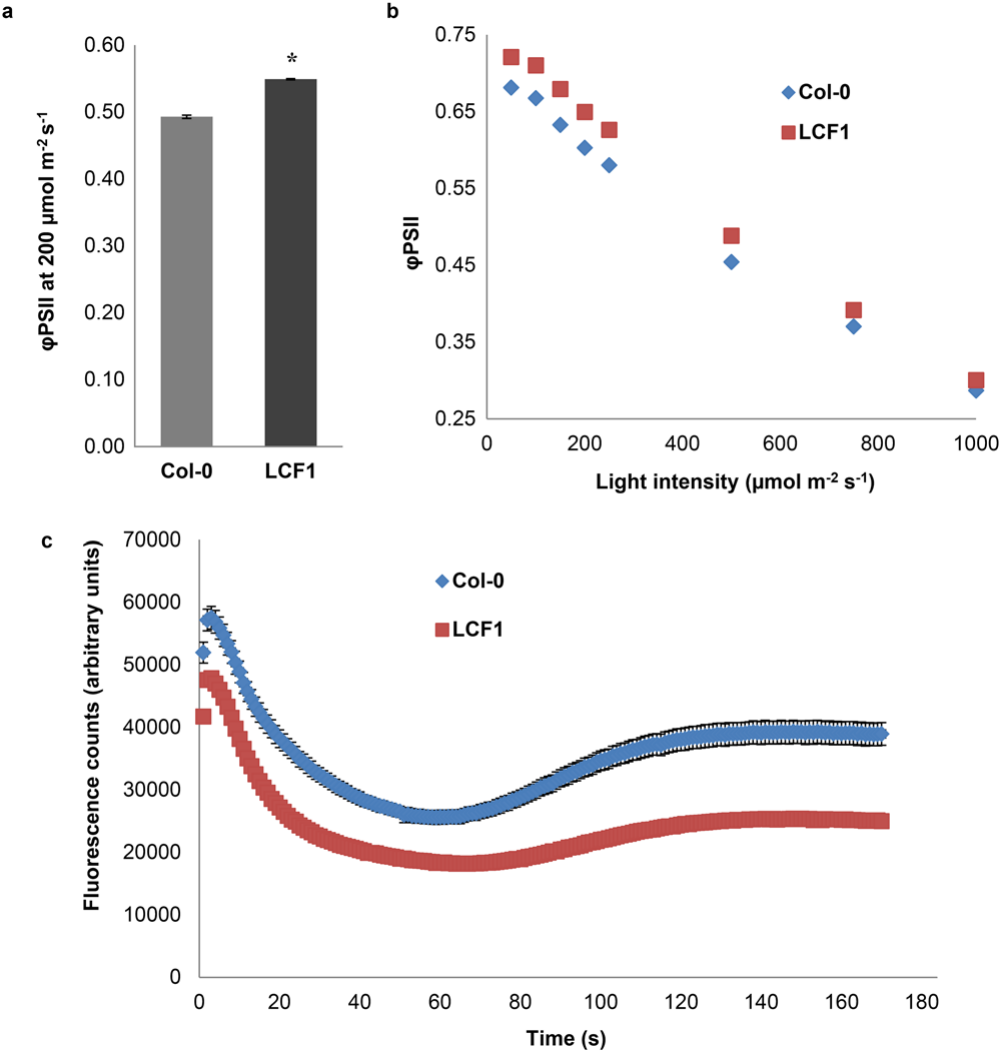
Quantification of the operating efficiency of Photosystem II (φPSII) of Col-0 and LCF1 plants using CF imaging. (**a**) Quantification of φPSII at standard light conditions of 200 μmol m^−2^ s^−1^ (14 days after germination; 30 s adaptation steps to the actinic light). (**b**) Quantification of φPSII at a range of different light intensities (19 days after germination; 15 minute adaptation steps to the actinic light). Asterisks (*) represent a significant difference with Col-0 (*p* < 0.05). Error bars represent SEM values (n = 6 per genotype) and are visible only when exceeding the data points. (**c**) Chlorophyll fluorescence induction traces of Col-0 plants and LCF1 plants. Plants were dark adapted at 14 days after germination for ≥30 min and placed in the imaging chamber. Subsequently, actinic light of 50 μmol m^−2^ s^−1^ was switched on and the counts of fluorescence were quantified. Error bars represent SEM values (n = 12 per genotype) and are visible only when exceeding the data points.

The φPSII of LCF1 plants was typically 10–15% higher than that of Col-0 plants at 200 μmol m^−2^ s^−1^ of actinic light (Fig. 3a) at every time point in rosette development. As our initial CF measurements were only performed at standard light conditions (200 μmol m^−2^ s^−1^ PAR), we also investigated the response of LCF1 plants to other light intensities. To this end, φPSII of Col-0 and LCF1 plants was quantified at a range of different light intensities with long adaptation steps to the actinic light, to ensure that the physiological responses of the plants to the changes in light intensity were reflected by φPSII. LCF1 plants had a significantly higher φPSII than Col-0 at every light intensity (Fig. 3b). As we had observed low steady state CF levels in LCF1 (Fig. 1), we were also interested in the dynamics of CF induction, the Kautsky effect^18^. To this end, induction was examined by quantifying the fluorescence of dark adapted Col-0 and LCF1 plants upon illumination with 50 μmol m^−2^ s^−1^ actinic light. Although the time resolution of our CF imaging setup did not allow for quantification of the Kaustky effect on a micro to millisecond time scale, the fluorescence induction kinetics of LCF1 were clearly different from those of Col-0 (Fig. 3c). The CF of LCF1 was consistently lower over the whole course of both the fast and slow transients of induction (referred to in literature as the Origin, Peak, Steady state (OPS) transient and the Steady State, Maximum, Terminal state (SMT) transient, respectively^19^), which can both be observed at a timescale of seconds. This suggested that LCF1 plants have higher photochemical quenching activity and more electron transport than Col-0 plants, resulting in a substantial decrease in CF from the origin of induction onwards.

**Figure 4:**
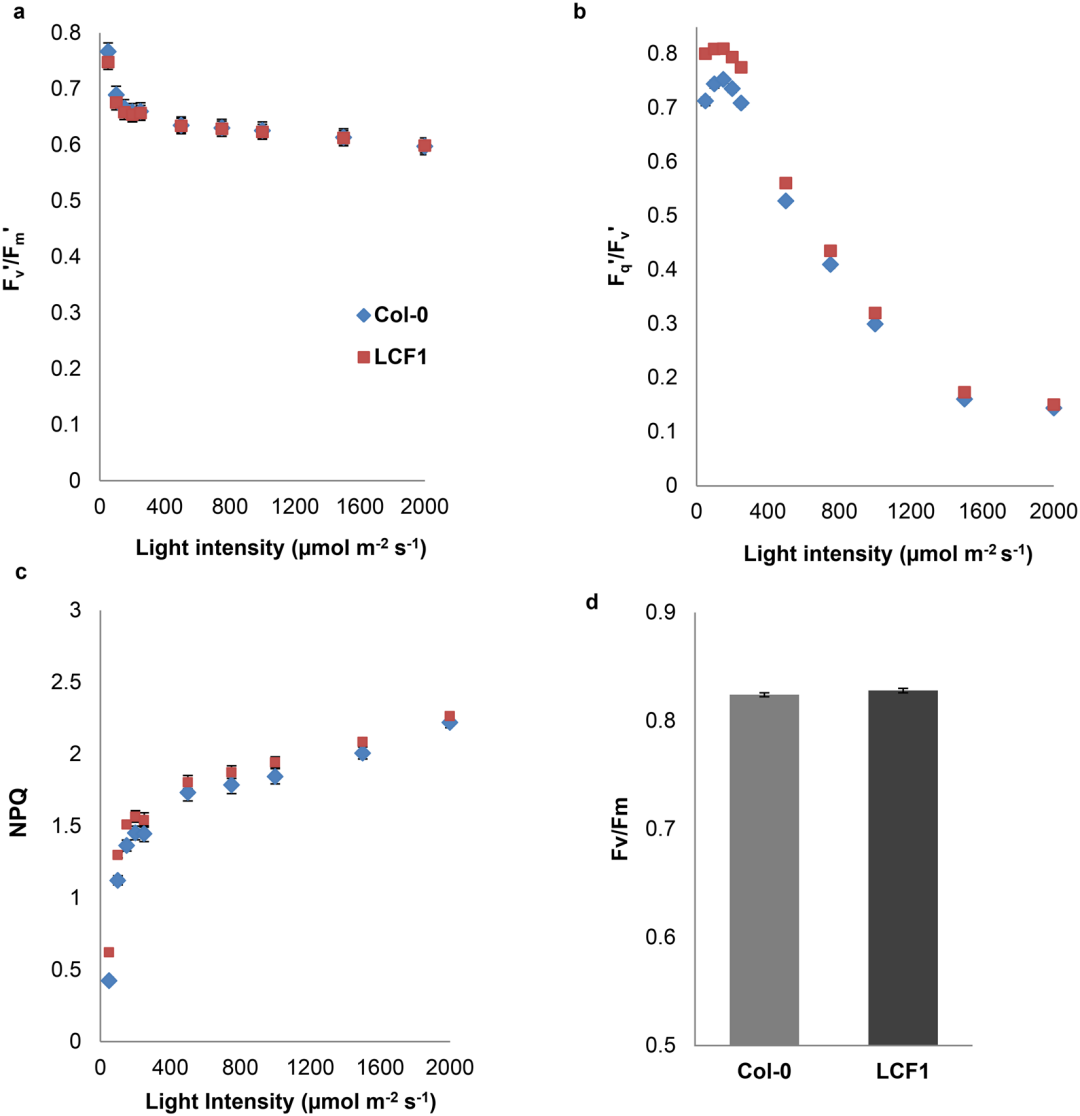
Quantification of (**a**) the maximum efficiency of Photosystem II in the light (F_v_′/F_m_′), (**b**) the coefficient of photochemical quenching (F_q_′/F_v_′) and (**c**) NPQ of Col-0 and LCF1 plants at different light intensities (19 days after germination). Response curves were made by adapting the plants to every actinic light intensity for 30 s, followed by a saturating pulse. (**d**) Quantification of the maximum quantum efficiency of PSII photochemistry (F_v_/F_m_) of dark- adapted Col-0 and LCF1 plants (19 days after germination). Error bars represent SEM values (n = 12 per genotype) and are visible only when exceeding the data points.

To further examine the role of photochemical quenching in the high φPSII and low CF phenotype of LCF1, light response curves were made for both the maximum efficiency of PSII in the light (F_v_′/F_m_′) and the PSII efficiency factor (F_q_′/F_v_′), which is a measure for the fraction of all PSII reaction centers that are open and thereby relates the maximum efficiency to the operating efficiency φPSII^8^. An increase in the maximum efficiency of PSII in the light might mean that LCF1 has intrinsically more efficient PSII reaction centers, whereas an increase in photochemical quenching is in line with a larger fraction of PSII reaction centers performing photochemistry in LCF1. There were no significant differences in the F_v_′/F_m_′ values of LCF1 compared to Col-0 (Fig. 4a), but the F_q_′/F_v_′ values were significantly higher (Fig. 4b). In addition, there were no statistically significant changes in NPQ (Fig. 4c) and F_v_/F_m_ (Fig. 4d). Altogether, these results demonstrated that the increase in φPSII of LCF1 plants is due to an increase in the electron transport of PSII reaction centers.

### The increase in φPSII and the low levels of chlorophyll fluorescence of LCF1 are due to a shift in PSII-PSI ratio

All of the CF measurements described above were performed on LCF1 plants that were performing steady state photosynthesis at non-stressful light conditions. However, these conditions are not representative of field conditions, where plants are exposed to a range of light conditions, also fluctuating between low and high intensity. To examine the response of LCF1 plants to these biologically more relevant conditions, Col-0 and LCF1 plants were exposed to either low (20 μmol m^−2^ s^−1^), high (1000 μmol m^−2^ s^−1^), very high (2000 μmol m^−2^ s^−1^) or fluctuating (50 μmol m^−2^ s^−1^ for 5 min, followed by 500 μmol m^−2^ s^−1^ for 1 min) actinic light intensities for a period of 1 h, before, during and after which φPSII was quantified every 15 min (Fig. S2). From these response curves it could be concluded that φPSII was consistently higher in LCF1 compared to Col-0, regardless of the light intensity. The relative increase in φPSII during light stress treatment compared to Col-0 varied from approximately 6% to 10% (Fig. S2). LCF1 plants were also able to either partially or almost fully recover from every light stress treatment when they were again exposed to 200 μmol m^−2^ s^−1^ of actinic light (Fig. S2).

**Figure 5:**
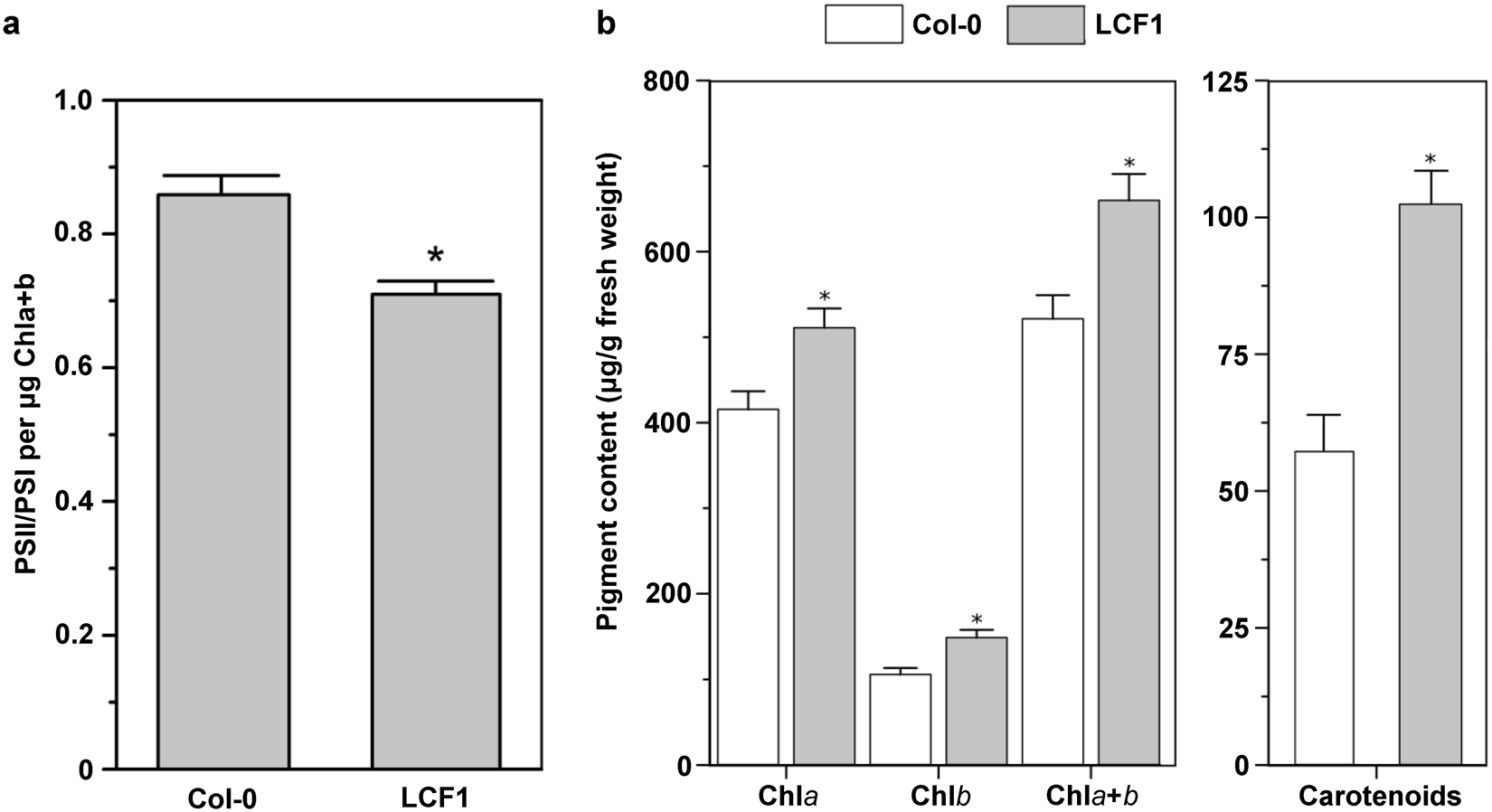
(**a**) The PSII-PSI ratio of Col-0 and LCF1 plants at 28 days after germination, determined by fluorescence spectrophotometry. Error bars represent SEM values (n = 7 per genotype). (**b**) Photosynthetic pigment content of leaves of Col-0 and LCF1 plants. The chlorophyll and total carotenoid contents were determined spectrophotometrically. Asterisks (*) represent a significant difference with Col-0 after one-way ANOVA (*p* < 0.05). Error bars represent SEM values (n = 7 per genotype).

To further investigate the high φPSII of LCF1, we determined the PSII:PSI ratio of Col-0 and LCF1 plants. This ratio was significantly lower for LCF1 (0.71 ± 0.07) than for Col-0 (0.86 ± 0.10) at *p* < 0.001 (Fig. 5a). These observations are in line with the notion that a lower PSII:PSI ratio points towards a higher quantum yield of photosynthesis^20^. As CF levels are for the largest part attributable to PSII fluorescence^8^, a lower PSII:PSI ratio can thus also explain why LCF1 exhibits lower CF, as electron transfer capacity through PSI downstream of PSII will not be limiting. These observations were corroborated by changes in photosynthetic pigment composition, with LCF1 plants having 41% more chlorophyll *b* (Fig. 5a) and 79% more carotenoids (Fig. 5b) than Col-0 on a fresh weight basis. LCF1 plants also had 23% more chlorophyll *a* than Col-0 (Fig. 5a), indicating an increase in the size of PSII antennae^21^.

### LCF1 plants accumulate more starch despite having reduced growth

**Figure 6:**
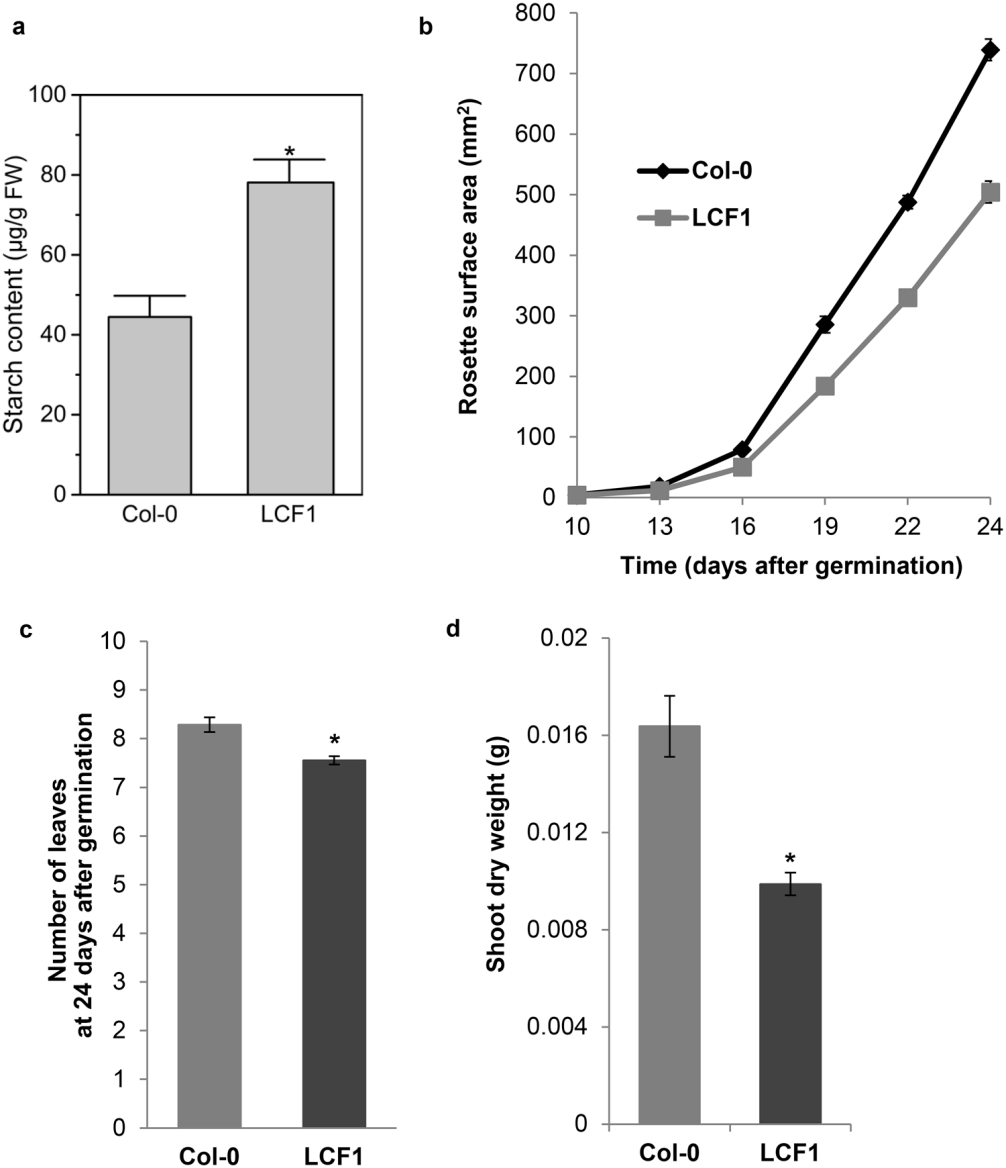
(**a**) Starch content of Col-0 and LCF1 plants at 28 days after germination (n = 3 per genotype). Starch concentration was determined through enzymatic digestion with α-amylase and α-amyloglucosidase, followed by the quantification of glucose concentration by spectrophotometry. Asterisk (*) represents a significant difference with Col-0, as determined by one-way ANOVA analysis (p < 0.01). (**b**) Growth curves of Col-0 and LCF1 plants. Error bars represent SEM values (n = 36 for Col-0, n = 99 for LCF1), and are only visible when exceeding the data points. (**c**) Number of true leaves with discernable petioles at 24 days after germination. Error bars represent SEM values (n = 35 for Col-0, n = 103 for LCF1. (**d**) Dry weight of Col-0 and LCF1 shoots at 24 days after germination. Error bars represent SEM values (n = 24 for Col-0, n = 69 for LCF1). Asterisks (*) represent a significant differences with Col-0 (*p* < 0.05).

As LCF1 has high φPSII, it might be expected to have increased overall photosynthetic performance and to accumulate more biomass than Col-0 plants. We firstly investigated whether LCF1 plants accumulated more starch than Col-0. Indeed, LCF1 was found to have 76% more starch available in the rosette leaves compared to Col-0 (Fig. 6a). These data are well in line with the observation of a strong correlation between high linear electron transport and elevated carbohydrate content that was reported recently for the Arabidopsis *hpe1* mutants^11^. In addition, we quantified the rosette surface area (RSA) of Col-0 plants and LCF1 plants throughout development. RSA was used as a proxy for biomass, because these two parameters are strongly correlated in Arabidopsis^22^. In contrast with the high level of starch, we found LCF1 plants to be consistently smaller throughout development than Col-0 (Fig. 6b). LCF1 plants had an approximately 32% smaller RSA than Col-0 plants at 24 days after germination (*p* < 0.001), which could partly be attributed to a decrease in the number of leaves (Fig. 6c). In concurrence with these observations, LCF1 plants accumulated significantly less dry weight (Fig. 6d) than Col-0 plants.

**Figure 7:**
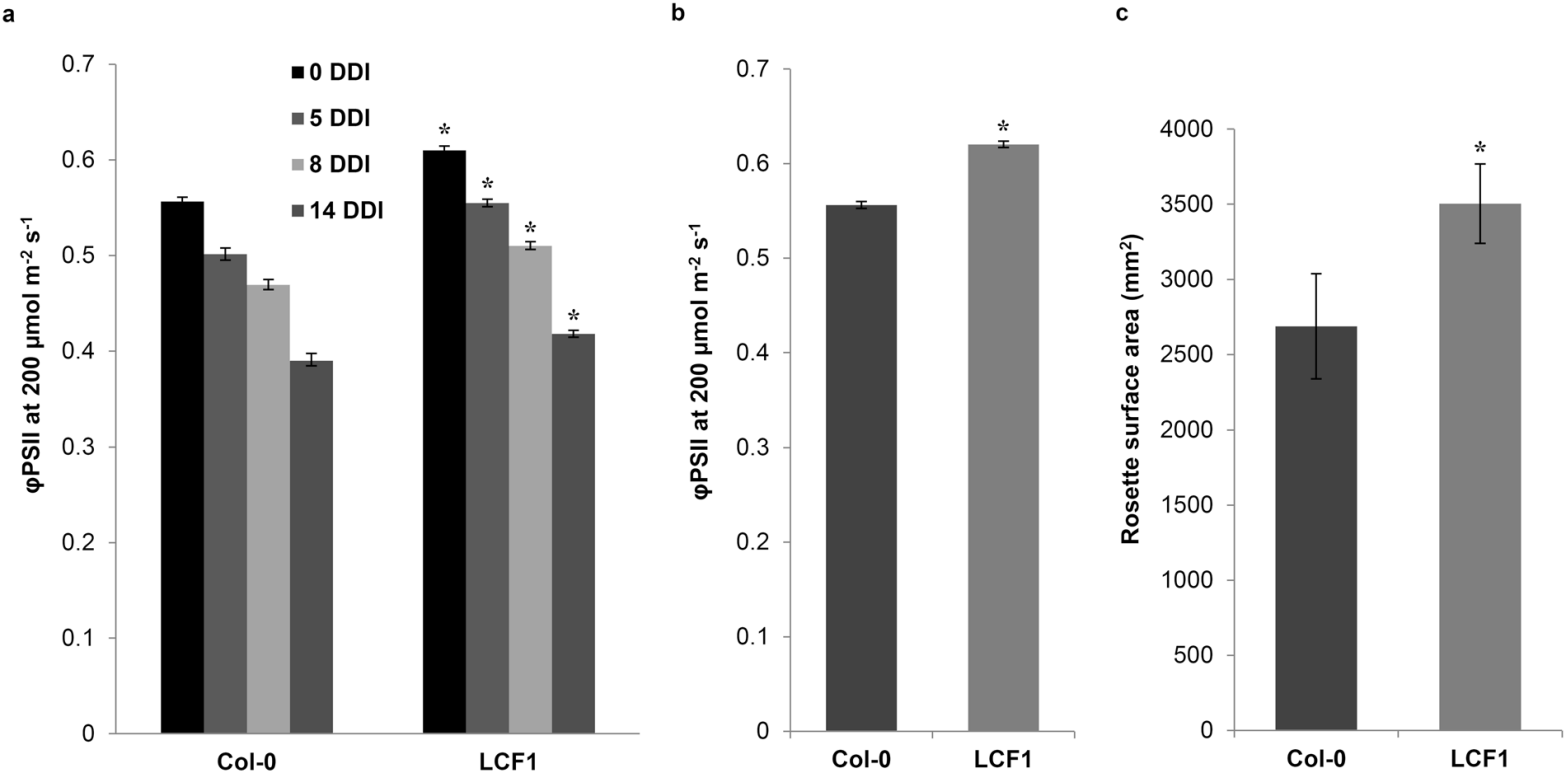
Dark induced senescence of Col-0 and LCF1 plants. Plants were initially grown at standard light conditions, and were transferred to complete darkness at otherwise unchanged conditions at 21 days after germination. (**a**) φPSII measurements after 0, 5, 8 and 14 days of dark incubation (DDI), respectively. (**b**) Quantification of φPSII after 14 days of recovery in the light. (**c**) Quantification of rosette surface area after 14 days of recovery in the light. Asterisks (*) represent a significant difference with Col-0 (*p* < 0.05 for φPSII values and *p* < 0.1 for rosette surface area values). Error bars represent SEM values (n = 7 per genotype).

To examine whether the growth reduction also occurred at light conditions other than standard conditions (12 h photoperiod at 200 μmol m^−2 ^s^−1^ PAR light; mercury lamps), Col-0 and LCF1 plants were grown at short day and low light conditions (8 h photoperiod at 50 μmol m^−2^ s^−1^ PAR light; fluorescent tubes), thereby reducing the irradiance on the plants and lowering photosynthetic rate^23^. At these conditions, there were no significant differences between LCF1 and Col-0 in terms of RSA (Fig. S3A and B) and biomass (Fig. S3C). However, when they were supplemented with 2000 ppm of CO_2_ to stimulate photosynthesis^24, 25^, there was again a reduction in growth over time (Fig. S3D). Taken together, these observations indicated that the growth of LCF1 plants is negatively correlated with photosynthetic rate. This was further investigated by first growing Col-0 and LCF1 plants under standard light conditions up till 21 days after germination and then transferring them to complete darkness, a treatment known to induce senescence, accompanied by large-scale pigment degradation and depletion of photosynthetic carbon^26^. Regardless of the number of days of dark incubation, LCF1 plants maintained a significantly higher φPSII than Col-0 plants (Fig. 7a), and regained their φPSII values from before dark-induced senescence after 14 days of recovery in the light (Fig. 7b). Remarkably, LCF1 plants had a significantly larger RSA than Col-0 plants after recovery in the light (Fig. 7c), indicating that the normal growth impediment of LCF1 plants was alleviated after the period of darkness and that the higher φPSII now correlated with enhanced growth.

## Discussion

In this study we described the isolation and phenotypic characterization of the novel Arabidopsis mutant line LCF1. Genetic analysis of LCF1 showed that the mutation causative for high φPSII and low CF was recessive and closely linked to a single T-DNA insertion within the *MORC2* gene (At4g36280). The enhanced photochemical properties of LCF1 were combined with a significant decrease in PSII:PSI ratio and an increase in carotenoid content. While LCF1 plants had stunted growth at standard conditions, they accumulated relatively more starch. In addition, the growth of LCF1 plants was restored to wild type levels at low light and short day conditions, and could be enhanced after a period of dark induced senescence.

To our knowledge, no other screens for Arabidopsis mutants with φPSII as a selection criterion have been reported in literature. There are, however, a large number of publications available which describe forward genetic screens using other CF parameters as selection criteria. Most of these screens have yielded mutants with high CF phenotypes, which are very easily recognizable^27–31^. The most extensive screen for Arabidopsis mutants with HCF phenotypes was described by Meurer *et al*. in 1996^27^, and yielded 34 recessive *hcf* mutants from a population of 7700 ethyl methane sulfonate (EMS)-mutagenized Arabidopsis plants. We have not encountered any conspicuous mutants with HCF phenotypes in our library of 4278 plant lines, which is likely due to the fact that most of the described *hcf* are partial loss-of-function mutants with amino acid substitutions in core photosynthesis proteins^27^ rather than full knock-out mutants or mutants producing truncated versions of these proteins, which are likely to be lethal.

The only other mutants with low CF and high φPSII like LCF1 are the recently described *hpe1* knock-out mutants^11^, which were isolated from a library of more than 50,000 insertion mutants harboring enhancer tagging constructs^32–34^ using F_v_/F_m_ as a selection criterion. The CF characteristics of the *hpe1* mutants and LCF1 are very similar, having low CF, as well as high φPSII and F_q_′/F_v_′ (also known as qP)^11^. However, marked differences exist. While *hpe1* plants have defective NPQ and higher F_v_/F_m_, LCF1 plants have wild type NPQ levels and F_v_/F_m_, the latter difference meaning that LCF1 plants could not have been isolated by the group of Jin *et al*. using F_v_/F_m_ as a selection criterion^11^. In principle, screening using F_v_/F_m_ as a selection criterion also allows for the isolation of mutants with enhanced photochemistry, as it requires dark adaptation of the plants implying that differences in light-driven physiology of photosynthesis might not be found. The fact that we have not isolated *hpe1* mutants in the present study could very well be due to the fact that the library screened by *Jin et al*.^11^ to isolate *hpe1* was ten-fold larger than the 3F-VP16 library. As an additional difference, while LCF1 plants displayed reduced growth at standard light conditions, *hpe1* plants had enhanced growth^11^. Also the molecular genetic data underlying the phenotypes are different; *HPE1* (At1g70200) encodes a chloroplast protein involved in the splicing of plastid RNAs, whereas the mutation causal for LCF1 is genetically linked to a T-DNA insertion in *MORC2* (At4g36280), encoding an ATPase of the Microrchidia (MORC) family which is active in the nucleus^35^. The annotated Arabidopsis genome does not contain any *HPE1* homologues in the vicinity of the *MORC2* locus at the top of chromosome 4 to which the phenotypes of LCF1 could be genetically linked.

Although the phenotype of LCF1 is genetically linked to the insertion of a 3F-VP16 encoding T-DNA construct in the *MORC2* gene (Fig. 2), we did not find any evidence that it is caused by a simple recessive knock-out mutation. None of 15 SALK lines containing T-DNA insertions in the *MORC2* locus produced plants with an LCF1 phenotype. Moreover, dedicated gene constructs aimed at complementing the LCF1 phenotype failed to do so (Fig. 2d). We found that mRNA corresponding to the 3F-VP16 transcription factor encoded by the T-DNA insert in *MORC2* of LCF1 was expressed (Fig. 2c and e), but when the SALK *MORC2* knock-out lines were transformed with the 3F-VP16 encoding construct isolated from LCF1 we did not find any transformants with an LCF1(-like) phenotype. Altogether, these findings might indicate that the 3F-VP16 expression construct specifically required the *morc2* mutant allele as found in LCF1 to exert its effect, an insertion allele which still allows for expression of the 5′ part of the MORC2 mRNA (Fig. 2e). Alternatively, it might be that the LCF1 phenotype is caused by an additional recessive mutation close to the T-DNA insertion. To discriminate between these possibilities, LCF1 plants were crossed with several of the SALK *MORC2* insertion lines, with the expectation that at least some of the different F1 plants would now show an epistatic effect of the LCF1 *morc2* allele in case that it would contribute to the phenotype. If not, F1 should have the normal photosynthetic parameters observed in the different SALK lines. However, despite various efforts and in contrast to control crossings with Col-0 plants, none of these crosses yielded any F1 seeds. We are therefore tempted to hypothesize that LCF1 plants express a truncated MORC2 protein which functions as a novel epigenetic regulator of *MORC2* target loci, and which in combination with highly insertion locus specific expression of the 3F-VP16 fusion results in the phenotype of LCF1. As *AtMORC2* encodes an ATPase of the Microrchidia (MORC) family that in plants and animals is involved in the large scale epigenetic transcriptional silencing of transposons through the condensation of heterochromatin^35^, it is conceivable that chromatin interactions of the truncated protein form the basis of the LCF1 phenotype. However, given the experiments which have already been performed it will be very challenging to verify this hypothesis.

As mentioned above, LCF1 plants display a combination of high φPSII and low chlorophyll fluorescence, which has recently only been reported for the *hpe1* knock-mutants^11^. To our knowledge, LCF1 is the first Arabidopsis mutant with a high φPSII and low CF likely due to an intrinsic decrease in PSII:PSI ratio. Interestingly, the increase in φPSII of LCF1 at standard light conditions (Figs 1a and 3a) is proportional to the decrease in PSII:PSI ratio (Fig. 5a), corroborating the notion that the PSII:PSI ratio is a determining factor for photochemical efficiency^20^. Combined with the fact that LCF1 has the same F_v_/F_m_ as Col-0, it is most likely that although both plant lines have equally high maximum efficiencies of PSII photochemistry, PSII reaction centers of LCF1 have a higher linear operational electron transport rate than Col-0 due to the availability of relatively more PSI reaction centers in the light. This would also explain why LCF1 plants have much lower CF than Col-0 over the whole course of CF induction irrespective of the phase of induction (Fig. 3c), and are able to adapt better to extreme light conditions (Fig. S2). In terms of φPSII photochemistry, LCF1 plants thus appear to outperform the normal high light adaptation of Col-0 plants, possibly not solely relying on the reallocation of antennae from PSII to PSI by means of state transitions^36^.

As LCF1 has substantially higher φPSII than Col-0, one might expect it to have a correspondingly higher overall rate of photosynthesis and, therefore, to accumulate more biomass. However, LCF1 displayed stunted rather than enhanced growth compared to Col-0 at standard growth conditions. There is considerable debate about whether or not more efficient photosynthesis necessarily results in an increase in growth and yield^25^. Obviously, sugar metabolism and transport are both of great influence to plant growth^37^, just as the extent to which starch is made available for metabolism and towards which organs carbohydrates are partitioned. In particular, the accumulation of trehalose-6-phosphate, which is a metabolic precursor of glucose, has been reported to negatively impact growth upon an excessive accumulation of starch^37^. As LCF1 accumulates much more starch than Col-0 (Fig. 6a), it could be envisaged that a corresponding accumulation of trehalose-6-phosphate is responsible for the feedback inhibition of the growth of LCF1. Such a scenario might explain why dark-grown LCF1 plants, which are devoid of starch, can grow faster than Col-0 plants upon adaptation to light (Fig. 7c). The finding that LCF1 plants grown at low light intensity and short day, thus protected from excessive starch accumulation, grew equally well as Col-0 plants despite having higher φPSII (Figs 3b and S4) seems to further corroborate the idea of a starch related feedback mechanism for growth. Moreover, when allowing for more efficient carbon fixation by supplementing reduced light conditions with additional CO_2_, growth of LCF1 plants was again compromised compared to Col-0 plants (Fig. S3D). These observations indicate that LCF1 plants are sensitive to feedback inhibition downstream of photosynthetic carbon fixation, while lowering photosynthetic rate allows growth to be rescued.

In conclusion, by screening a library of T-DNA insertion mutants with φPSII as a selection criterion we have isolated the interesting novel mutant LCF1. This shows that it should be possible to isolate novel plant lines with enhanced photosynthetic properties using forward genetic screens. The genetics underlying the photosynthetic properties of LCF1 proved to be complex, but LCF1 phenotype indicated that it should be possible to develop plants which perform significantly better under adverse light conditions, such as low light intensity.

## Materials and Methods

### Growth conditions

All plants in this study were grown on soil in a climate-controlled growth chamber at a constant temperature of 20 °C, 70% relative humidity, and at a light intensity of approximately 200 μmol m^−2^ s^−1^ of photosynthetically active radiation (PAR) from mercury lamps at a 12 h photoperiod, unless otherwise specified. These light conditions are referred to as ‘standard light conditions’. Seeds were always stratified on soil for 3–4 days at 4 °C prior to being placed in the growth chamber. Plants grown at a low light intensity were firstly grown at standard light conditions, and were transferred to a growth cabinet with fluorescent tubes at a PAR light intensity of approximately 50 μmol m^−2^ s^−1^ at 14 days after germination.

### Plant material and genome interrogation library construction

The Arabidopsis accession Columbia-0 (Col-0) was used as a wild type in this study. All experiments with LCF1 were conducted with the T3 progeny of the originally isolated T2 LCF1 individual. SALK T-DNA insertion knock-out lines for the genomic locus At4g36280 (*MORC2*) were obtained from the Nottingham Arabidopsis Stock Centre (Table 1). A library of T-DNA constructs encoding fusions of three zinc fingers (3Fs) to the transcriptional activator VP16 was constructed in the binary vector pRF-VP16-Kana as described previously^13^. Col-0 plants were transformed with these constructs using the floral dip method^38^. Primary transformants (T1 generation) were selected on MA medium containing 35 μg/mL kanamycin, and were transferred to soil after approximately 2 weeks. The primary transformants were then raised, and their seeds were harvested. Due to plant loss and infertility of a fraction of the plants, not all plants set seeds. Seeds of five or less primary transformants (T2 seeds) originating from the same 3F pool were combined and stored in a single seed bag. These bags were named ‘five-bags’. The genome interrogation library consisted of the T2 seeds of 4278 plant lines in total representing approximately 3500 3F-VP16 encoding constructs, and were stored in a total of 1034 five-bags.

### Library screening for φPSII mutants

Approximately 20 seeds from every five-bag were sown. At 14 days after germination the φPSII of the plants was quantified at 200 μmol m^−2^ s^−1^ of actinic light (the same intensity of PAR light as in the growth chamber) using a Technologica CF Imager (Technologica, Colchester, United Kingdom). The φPSII (F_q_′/F_m_′^8^) images were generated after 30 s of exposure to the actinic light with a 800 ms saturating pulse of 6226 μmol m^−2^ s^−1^ (maximal intensity).

### Genetic analysis of LCF1

For segregation analysis, LCF1 (♂) was crossed with Col-0 (♀). F1 individuals were selected for presence of the 3F-VP16 encoding T-DNA construct on half strength MS medium containing 50 μg/mL kanamycin. After 2–3 weeks on selection medium, resistant individuals were transferred to soil. The F2 seeds were harvested and φPSII of a population of F2 individuals was quantified as described above. The T-DNA copy number in the genome of LCF1 was examined using the Southern blot method^39^ on *Nco*I predigested genomic DNA isolated with the CTAB extraction protocol^40^. As a probe, DIG-labelled PCR product was generated with the primer combination RPS5a FW and tNOS REV (Table 2) from LCF genomic DNA, using PCR DIG Labelling Mix (Roche). Detection of hybridization signal was performed with the DIG Wash and Block Buffer Set (Roche), according to the instructions provided by the manufacturer. The insertion site of the 3F-VP16 encoding T-DNA construct in the genome of LCF1 was mapped via TAIL-PCR^41^, using forward primers NOS1 FW, NOS2 FW and NOS3 FW (Table 2), respectively, for the three consecutive PCR reactions each with one of degenerative primers AD1, AD2 and AD3 (Table 2), respectively. TAIL-PCR products were cloned into the pJET Blunt cloning vector using the CloneJet PCR Cloning Kit (Thermo Scientific), and sequenced by Sanger sequencing (Macrogen Europe, Amsterdam, The Netherlands). The 3′ regions of the PCR products were BLASTed (http://blast.ncbi.nlm.nih.gov/Blast.cgi) for identification of the insertion locus. For the confirmation of insertion locus At4g36280 (*MORC2*), PCRs were performed using combinations of At4g36280 specific primers (FWa, FWb, REVa, REVb; Table 2) and combinations of At4g36280 and T-DNA specific primers (RPS5a FW, tNOS REV; Table 2).

**Table 2:** Primers used for the genetic analysis of LCF1.

Primer name	Sequence (5′-3′)	Restriction site underlined
NOS1 FW	GATTGAATCCTGTTGCCGGTCTT	
NOS2 FW	GCATGACGTTATTTATGAGATGG	
NOS3 FW	CGCAAACTAGGATAAATTATCGC	
AD1	NTCGASTWTSGWGTT	
AD2	NGTCGASWGANAWGAA	
AD3	WGTGNAGWANCANAGA	
At4g36280 FWa	ACGGAGTAGTAGGAGGAAGAGA	
At4g36280 FWb	TTGGAAGGCGGGAGATTACT	
At4g36280 REVa	CTTTTTCAACCTCGCCTCCA	
At4g36280 REVb	TGTAGGTTGTGGGTTGAGCTG	
RPS5a FW	GCCCAAACCCTAAATTTCTCATC	
tNOS REV	CAAGACCGGCAACAGGAT	
MORC2 cDNA FW	AACCATGGCTCCTATGGCGAAAAATGCAG	NcoI
MORC2 cDNA REV	AAGGTCACCTAAGCTTGTTGCATCTCCT	BstEII
ATG6 FW	AGACACAGGTTGAACAGCCA	
ATG6 REV	GTATGCTTCCACGTCCCTCG	
SCAMP5 FW	TCACCTACTTGATTCACAT TGGCT	
SCAMP5 REV	ATCAATTGCTGCAAGCACAC	
VP16 FW	ATTTACCCCCCACGACTCC	
VP16 REV	ACCACCGTACTCGTCAATTC	
At4g36270 FW	CCTATGATCCGATATGCAA	
At4g36270 REV	GTGGGTGGAAGATGGAAA	
MORC2Δ FW	GCCTCCTATGGCGAAAAA	
MORC2Δ REV	GTACTTGCTGATTCTCTTCCT	
MORC1 FW	GAAAAATTACACAGTCGCC	
MORC1 REV	CTATTAGAGATGCCAAGC	
MORC2 gene FW	AAACTAGTCTCAACCCACAACCACCT	SpeI
MORC2 gene REV	AAGGTCACCTCCACCGCCATCATCTT	BstEII
171 FW	ACTTGTTTGGTATTTGTCTC	
171 REV	AATTCTACGGATAAGTTCAG	
880 p1 FW	GGTTTCTCTCCTTTCTT	
1325 p1 REV	TCCTGACCAGTTTTTCT	

Nucleotide codes: ‘N’ is any of the four bases; ‘W’ is either A or T; ‘S’ is either G or C.

### Genetic mapping

To establish a population of plants for genetic mapping LCF1 (♂) was crossed with the Arabidopsis accession Landsberg erecta (Ler-0; ♀). The resulting F1 hybrids were allowed to set seeds (F2) and φPSII of a population of F2 seedlings was quantified at 200 μmol m^−2^ s^−1^ actinic light at 7 days after germination as described above. Genomic DNA was isolated from a single young leaf of individuals exhibiting the high φPSII phenotype using a quick DNA extraction protocol^42^. The individuals were then genotyped by PCR analysis using primers 171 FW and REV (Table 2) for the indel marker 4-AL022141-9227 (http://amp.genomics.org.cn), which yields a 157 bp product for a Col-0 sequence and a 134 bp product for a Ler-0 sequence. The presence of wild type At4g36280 loci or loci with T-DNA inserts was determined by PCR analysis with the primer combination 880 FW and 1325 p1 REV (Table 2).

### Transformation with T-DNA construct reconstituted from LCF1

The 3F-VP16 encoding fragment was isolated from LCF1 by PCR using the T-DNA specific primer combination RPS5a FW and tNOS REV (Table 2). A reconstituted T-DNA construct was generated in the binary vector pRF-VP16-Kana^13^ using the 3F encoding sequence from this PCR product. Col-0 plants were transformed with the reconstituted T-DNA construct using the floral dip method^38^. Primary retransformants were selected by plating sterilized seeds on MA medium containing 35 μg/mL kanamycin, and transferred to soil after 2–3 weeks.

### RT-PCR and RT-qPCR analysis

Col-0 and LCF1 plants were ground to powder in liquid nitrogen with pistils and mortars at 35 days post germination. Total RNA was extracted from 50–100 mg of tissue powder of each individual using the RNeasy Plant Mini Kit (QIAGEN). RT-PCR reactions were performed using the OneStep RT-PCR Kit (Qiagen), with primer combinations specific for *MORC2* (MORC2 cDNA FW and REV), the reference genes *ATG6* and *SCAMP5* described by Hruz *et al*.^43^ (ATG6 FW and REV; SCAMP5 FW and REV), and for the 3F-VP16 fusion encoded by the T-DNA in LCF1 (VP16 FW and REV). For RT-qPCR analysis, first strand cDNA synthesis was performed using the iScript Select cDNA Synthesis Kit (BIORAD), and the PCR reactions were prepared using the SYBR Green PCR Master Mix (Applied Biosystems). The RT-qPCR reactions were performed using the CFX96 Touch Real-Time PCR Detection System (BIORAD), using primer combinations specific for At4g36270 (At4g36270 FW and REV), the 5′ end of *MORC2* expected to still be expressed in LCF1 (MORC2Δ FW and REV), *MORC1* (MORC1 FW and REV), and combinations specific for *ATG6*, *SCAMP5* and the *3F-VP16* construct as described above for RT-PCR. Expression values were normalized to the average of the expression values of both *ATG6* and *SCAMP5*. The sequences of all primers are listed Table 2.

### Complementation assay

The full length genomic *MORC2* (At4g36280) sequence (including promoter and terminator sequences) was amplified by PCR from genomic DNA of Col-0 isolated with the CTAB extraction protocol^40^, using the primer combination MORC2 gene FW and MORC2 gene REV (Table 2). The PCR fragment was ligated into the binary vector pCAMBIA3301 (CAMBIA, Canberra, Australia) predigested with XbaI and BstEII. The resulting binary vector was designated pCAMBIA3301A. The cDNA of At4g36280 was isolated from a root cDNA library^44^ using the primer combination MORC2 cDNA FW and MORC2 cDNA REV (Table 2). The PCR fragment was ligated into the binary vector pCAMBIA3301 predigested with NcoI and BstEII, thereby placing the cDNA under control of the NOS promoter and terminator sequences. This binary vector was designated pCAMBIA3301C. The plasmids pCAMBIA3301, pCAMBIA3301A and pCAMBIA3301C were mobilized to *Agrobacterium tumefaciens* strain LBA1100^45^ as described previously^14^. Col-0 and LCF1 plants were transformed with the constructs using the floral dip method^38^. Primary transformants were selected by plating the resulting seeds pools on MA medium containing 25 μg/mL PPT, and were transferred to soil after approximately 2 weeks. At 28 days after germination the φPSII of all primary transformants was quantified at 200 μmol m^−2^ s^−1^ of actinic light as described above.

### Chlorophyll fluorescence measurements

The quantification of φPSII was performed as described above, with adaptation steps to the actinic light lasting 30 to 60 s, depending on the number of plants that had to be analyzed. For the quantification of φPSII at different light intensities (‘slow’ light response curves), plants were adapted to every light intensity for 15 min instead of 30 s as described above. The maximum quantum efficiency of PSII photochemistry (F_v_/F_m_) was also quantified using the Technologica CF Imager (Technologica, Essex, United Kingdom) on dark adapted (≥30 min) plants. F_m_ images were generated with a 800 ms saturating pulse of 6226 μmol m^−2^ s^−1^ (maximal intensity). ‘Fast’ light response curves of φPSII were made on dark adapted (≥30 min) plants after F_v_/F_m_ quantification, thereby also allowing for the calculation of NPQ, F_q_′/F_v_′ and F_v_′/F_m_′ images, respectively. For the quantification of the Kautsky effect, dark adapted (≥30 min) plants were exposed to 50 μmol m^−2^ s^−1^ of actinic light, and the absolute counts of fluorescence were quantified for 3 min using the Technologica CF Imager. At the other light conditions, φPSII was quantified every 15 min for 1 h at 200 μmol m^−2^ s^−1^ of actinic light as described above, then conditions were switched to either low (20 μmol m^−2^ s^−1^), fluctuating (50 μmol m^−2^ s^−1^ for 5 min, followed by 500 μmol m^−2^ s^−1^ for 1 min), high (1000 μmol m^−2^ s^−1^) or very high (2000 μmol m^−2^ s^−1^) actinic light. φPSII was then quantified again every 15 min for 1 h (except for fluctuating light conditions, were φPSII was quantified after every 5 min of 50 μmol m^−2^ s^−1^ light and every 1 min of 500 μmol m^−2^ s^−1^), followed by a quantification of φPSII every 15 min for 6 h at 200 μmol m^−2^ s^−1^. The calculation of the CF parameters was performed with the average of all pixels in every rosette. All CF data were statistically analyzed using the heteroscedastic T-Test function of Microsoft Excel 2010 (assuming unequal variance between samples). A *p*-value of 0.05 was used as a threshold for significance.

### Analysis of pigment content and PSII-to-PSI ratio

Analysis of pigment content was performed on Col-0 and LCF1 plants at 28 days after germination. At the middle of the light period (t = 6 hours), shoots were harvested, frozen in liquid nitrogen and stored at −80 °C. The chlorophyll and total carotenoid contents were determined in dimethylformamide according to the method of Porra *et al*.^46^. Chloroplasts were isolated according to the protocol of Casazza *et al*.^47^. The chlorophyll concentration in the chloroplast isolates was determined according to the protocol of Arnon^48^. To obtain the PSII-to-PSI ratio, fluorescence emission spectra were obtained at 663 nm using a Cary Eclipse fluorescence spectrophotometer (Varian) at room temperature^49^. The peak at 680 nm in the fluorescence spectra corresponds to PSII emission and the pronounced shoulder at 730 nm corresponds to PSI emission. The PSII-to-PSI ratio was calculated as I_730nm_/I_680nm_. All data were statistically analysed using the one-way ANOVA function of OriginPro v2016 (OriginLab Corporation, Northampton, MA, USA). A *p*-value of 0.05 was used as a threshold for significance.

### Analysis of shoot starch content

Briefly, starch was extracted from the shoots of Col-0 and LCF1 plants (28 days after germination) with 80% ethanol and gelatinized at 95 °C, following the protocol of Smith and Zeeman^50^. The shoots were harvested at t = 6 h into the light period. Starch was then digested with α-amylase and α-amyloglucosidase at 37 °C. After digestion, the starch content was determined as released glucose with a hexokinase/glucose-6-phosphate dehydrogenase-based spectrometer assay at 340 nm. The data were statistically analysed using the one-way ANOVA function of OriginPro v2016 (OriginLab Corporation, Northampton, MA, USA). A *p*-value of 0.01 was used as a threshold for significance.

### Growth analysis

Photos were taken from the top of the plants with a fixed digital camera (Canon EOS 1100D) from 10 days after germination onwards and every three days. Using an ImageJ plugin, the intensity of the green channel of these RGB images was multiplied by two, both the red and blue channels were subtracted and the image was converted to a binary image using the ImageJ ‘Intermodes’ Threshold Method. The binary images were manually inspected to make sure that all the leaves of each individual plant were connected to each other as a rosette. In cases where leaves were not properly connected, they were connected manually to the rest of the rosette by a black line of two pixels in width. The surface area of each rosette was subsequently calculated in pixel^2^ using the ‘Analyze Particles’ function of ImageJ, and then converted to mm^2^ by multiplying this value by the mm^2^/pixel^2^ ratio of every RGB image separately. This ratio was calculated from the dimensions of the pots that the plants were growing in, as this value is constant for every individual throughout the experiment. At 24 days after germination, the leaves with clearly distinguishable petioles were counted manually and shoots were harvested for fresh weight determinations. Dry weight was determined after incubation of the shoots for 2 days at 60 °C. All growth parameter data were statistically analyzed using the heteroscedastic T-Test function of Microsoft Excel 2010 (assuming unequal variance between samples). A *p*-value of 0.05 was used as a threshold for significance.

### Analysis of the effect of dark induced senescence

The φPSII of Col-0 plants and LCF1 plants was quantified at 21 days after germination at 200 μmol m^−2^ s^−1^ of actinic light as described above. The plants were subsequently transferred to complete darkness at otherwise unchanged conditions. At 5 days of dark incubation (DDI), 8 DDI and 14 DDI, respectively, plants were temporarily adapted to 200 μmol m^−2^ s^−1^ of PAR light in the greenhouse for approximately 3 h and φPSII was quantified at 200 μmol m^−2^ s^−1^ of actinic light, as described above. At 14 DDI, plants were transferred back to standard light conditions. After 14 days of incubation in the light (now 49 days after germination), φPSII was quantified as described above. Rosette surface area data for all individuals were also obtained from the fluorescence images (in mm^2^). Data were statistically analyzed using the heteroscedastic T-Test function of Microsoft Excel 2010 (assuming unequal variance between samples). A *p*-value of 0.05 was used as a threshold for significance in the case of φPSII and 0.1 in the case of rosette surface area.

## Electronic supplementary material

Supplementary information
