# Supplementary material for: An Arabidopsis mutant with high operating efficiency of Photosystem II and low chlorophyll fluorescence
Source: Sci Rep. 2017 Jun 12;7:3314. doi: 10.1038/s41598-017-03611-1 (PMC5468348; doi:10.1038/s41598-017-03611-1)
Supplement: Supplementary file 1 — Supplementary information [file 41598_2017_3611_MOESM1_ESM.pdf]

An Arabidopsis mutant with high operating efficiency of Photosystem II and low chlorophyll fluorescence

Niels van Tol[1,2], Martijn Rolloos[1], Dieuwertje Augustijn[3], A. Alia[3], Huub J. de Groot[3],
Paul J.J. Hooykaas[1], and Bert J. van der Zaal[1*]

[1]Institute of Biology Leiden, Faculty of Science, Leiden University, Sylviusweg 72, 2333 BE, Leiden, The Netherlands
[2]BioSolar Cells, P.O. Box 98, 6700 AB Wageningen, The Netherlands
[3]Leiden Institute of Chemistry, Faculty of Science, Leiden University, Einsteinweg 55, 2333 CC, Leiden, The Netherlands
[*]Corresponding author

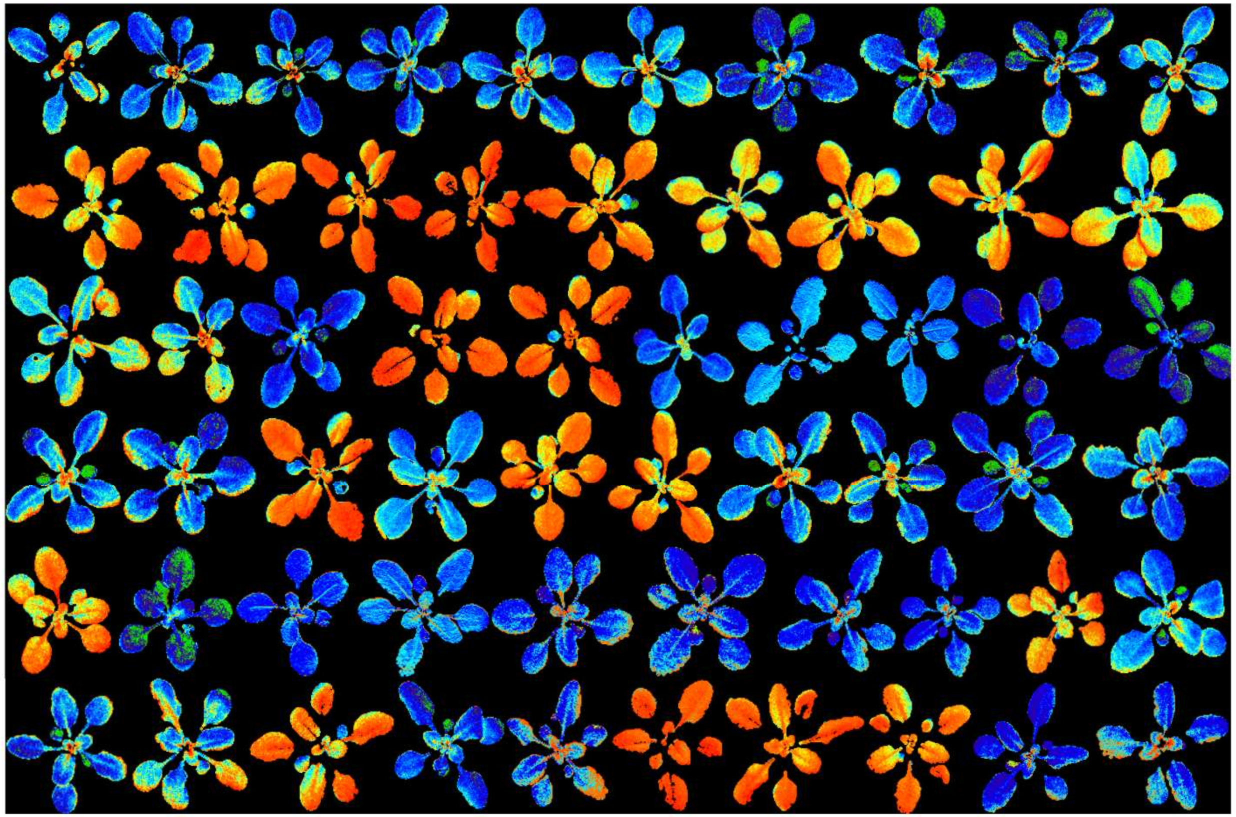

Fig. S1 False color φPSII images of 10 individuals each of the wild type Col-0, LCF1 (T3 generation), and four independent F2 progeny lines (F2,1-4) of a cross between LCF1 (pollen donor, ♂) and the wild type (♀). Plants were grown at standard light conditions, and φPSII of every individual was quantified at 200 µmol m^{-2} s^{-1} of actinic light. Orange colored individuals have the high φPSII phenotype; blue colored individuals have wild type φPSII values. Images do not provide a quantitative measure of φPSII.

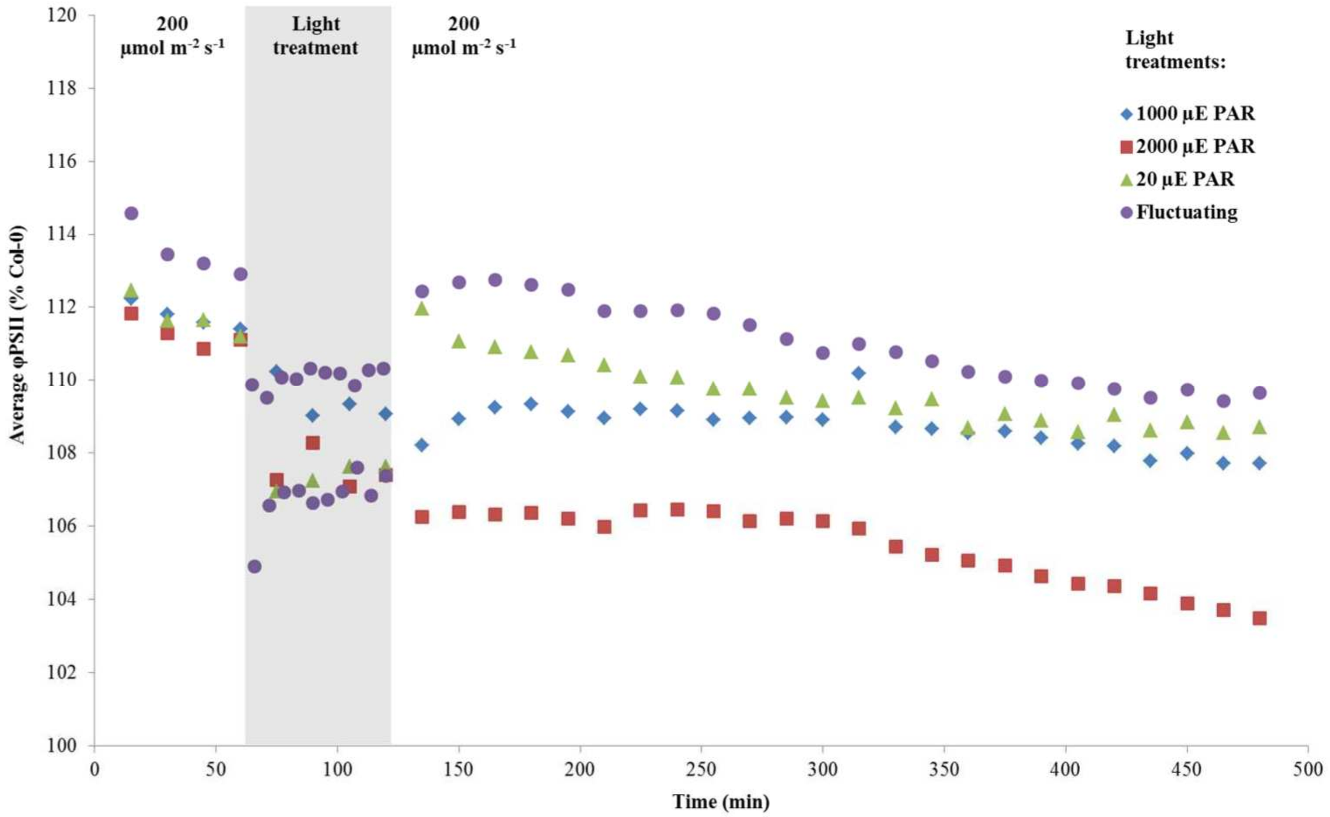

Fig. S2 The relative response of the operating efficiency of Photosystem II (φPSII) of LCF1 plants to light stress treatments compared to wild type Col-0 plants. The φPSII of LCF1 and Col-0 plants that were grown at standard light conditions of 200 µmol m^{-2} s^{-1} was quantified every 15 min for 1 h at 200 µmol m^{-2} s^{-1} of actinic light. Subsequently, as a light stress treatment, plants were exposed to actinic light of high intensity (1000 or 2000 µmol m^{-2} s^{-1}; 14 and 16 dpg respectively), low intensity (20 µmol m^{-2} s^{-1}; 21 dpg) or fluctuating intensity (50 µmol m^{-2} s^{-1} for 5 min, followed by 500 µmol m^{-2} s^{-1} for 1 min; 24 dpg), and φPSII was again quantified every 15 min for 1h (except for fluctuating light conditions, were φPSII was quantified after every 5 min of 50 µmol m^{-2} s^{-1} light and after every 1 min of 500 µmol m^{-2} s^{-1}). After the light stress treatment, standard light conditions were switched back on and φPSII was quantified every 15 min for 6 h. The response curves represent the average φPSII of LCF1 relative to that of the wild type (n ≥ 4 each).

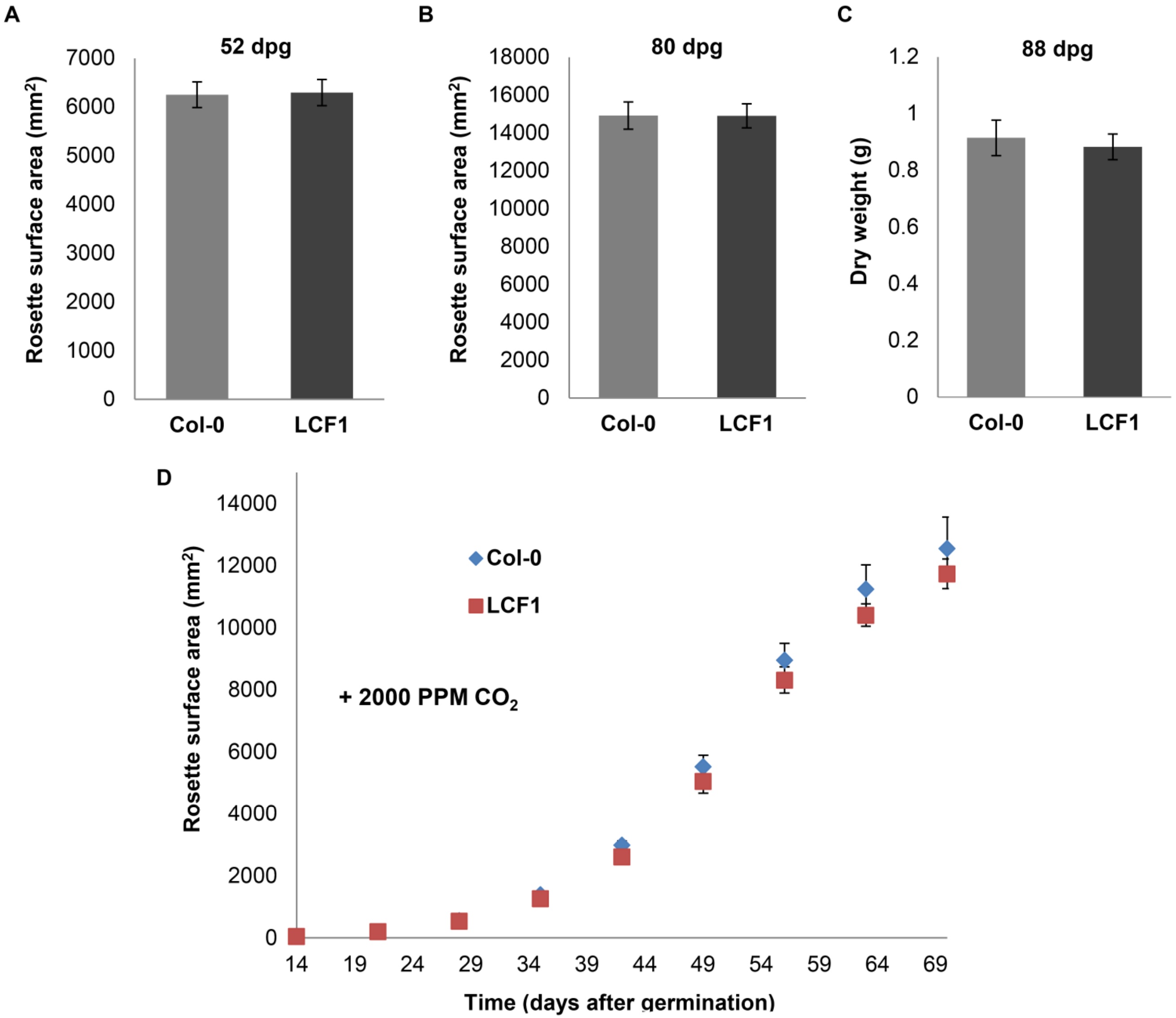

Fig. S3 Rosette surface area and biomass quantifications of wild type Col-0 and LCF1 plants grown at an 8 h photoperiod and a light intensity of 50 µmol m^{-2} s^{-1}. Plants were first grown at standard light conditions, and were transferred to the indicated conditions at 14 dpg. (**A**) Rosette surface area at 52 dpg. (**B**) Rosette surface area at 80 dpg. (**C**) Shoot dry weight at 88 dpg. (**D**) Growth curves of Col-0 plants and LCF1 plants grown at an 8 h photoperiod, a light intensity of 50 µmol m^{-2} s^{-1} and 2000 ppm CO_2. Plants were first grown at standard light conditions for 14 days and were then transferred to the indicated conditions. Error bars represent SEM values (n=6 per genotype).
